# Synthesis, characterization and in vitro effects of 7 nm alloyed silver–gold nanoparticles

**DOI:** 10.3762/bjnano.6.124

**Published:** 2015-05-27

**Authors:** Simon Ristig, Svitlana Chernousova, Wolfgang Meyer-Zaika, Matthias Epple

**Affiliations:** 1 Inorganic Chemistry and Center for Nanointegration Duisburg-Essen (CeNIDE), University of Duisburg-Essen, 45117 Essen, Germany

**Keywords:** cytotoxicity, gold, nanoalloys, nanoparticles, silver

## Abstract

Alloyed silver–gold nanoparticles were prepared in nine different metal compositions with silver/gold molar ratios of ranging from 90:10 to 10:90. The one-pot synthesis in aqueous medium can easily be modified to gain control over the final particle diameter and the stabilizing agents. The purification of the particles to remove synthesis by-products (which is an important factor for subsequent in vitro experiments) was carried out by multiple ultracentrifugation steps. Characterization by transmission electron microscopy (TEM), differential centrifugal sedimentation (DCS), dynamic light scattering (DLS), UV–vis spectroscopy and atomic absorption spectroscopy (AAS) showed spherical, monodisperse, colloidally stable silver–gold nanoparticles of ≈7 nm diameter with measured molar metal compositions very close to the theoretical values. The examination of the nanoparticle cytotoxicity towards HeLa cells and human mesenchymal stem cells (hMSCs) showed that the toxicity is not proportional to the silver content. Nanoparticles with a silver/gold molar composition of 80:20 showed the highest toxicity.

## Introduction

Over the last decades, noble metal nanoparticles have become a prominent subject in scientific studies due to their distinct physicochemical properties [1–2]. Apart from their catalytic and optical features, in particular, silver and gold nanoparticles have begun to play a major role in biochemistry, biology and medicine [3–5]. Silver nanoparticles are known to be highly toxic towards bacteria [6–8]. As a result, they are often employed as antibacterial agents in biomedicine or in consumer products [9–11]. Unfortunately, the therapeutic window for silver nanoparticles is rather narrow as silver nanoparticles are also toxic towards eukaryotic cells [11–12]. In contrast, gold nanoparticles are almost biologically inert (unless they are very small) [13] and therefore widely used in tumor therapy, for drug delivery, or in imaging applications [3,14–15].

In principle, alloyed nanoparticles of silver and gold can combine and utilize the physicochemical properties of both metals, for example, the optical properties of gold and the toxicity towards cells or bacteria of silver. In addition, very small nanoparticles (below about 2 nm) become autofluorescent [16–17]. There are several reports on different strategies to prepare alloyed silver–gold nanoparticles. Synthetic routes include wet chemical syntheses by co-reduction of gold and silver salts with citrate [18–19], NaBH_4_ [20] or starch and glucose [21], reduction with hydrazine in water-in-oil emulsions [22], sol–gel processes [23] or UV irradiation [24]. Recently, the generation of alloyed silver–gold nanoparticles by laser ablation was reported [25–27]. Alloying of presynthesized silver core/gold shell nanoparticles by refluxing with oleylamine [28] or ultrasonication of separate gold and silver nanoparticles [29] was also described.

Here, an aqueous co-reduction of silver nitrate and tetrachloroauric acid with a mixture of citrate and tannic acid was used to generate the alloyed nanoparticles. This synthetic route was previously used for the synthesis of pure gold and silver/gold 50:50 nanoparticles [30]. Interestingly, the addition of tannin, a polyphenolic biomolecule frequently used in the synthesis of gold nanoparticles [31], leads to a considerably faster reduction rate than citrate alone and also increases the colloidal stability of the resulting nanoparticles. We demonstrate the versatility of this synthetic route for the generation of alloyed nanoparticles using the silver/gold ratio as a size control over the nanoparticles, which can be achieved by variation of the amount of reducing agent. For further stabilization, the ligands can be easily exchanged without affecting the purity of the resulting dispersion. In this case, citrate and tannin were replaced by the frequently used stabilizer, poly(vinylpyrrolidone) (PVP). This ligand efficiently replaces the citrate, as previously demonstrated for gold nanoparticles [32]. A purification of the nanoparticles to remove the synthesis byproducts was achieved by multiple ultracentrifugation steps and did not affect the stability of the dispersions. The alloyed nanoparticles were characterized with respect to their physicochemical properties and their in vitro reaction.

## Results

### Nanoparticle characterization

Alloyed Ag/Au nanoparticles were synthesized similar to the previously reported synthesis of alloyed Ag/Au 50:50 nanoparticles [30]; however, higher amounts of the reducing agents (citrate and tannic acid) were used to generate nanoparticles with mean diameters of 6 to 7 nm. The primary ligands, citrate and tannic acid, were replaced by PVP after the synthesis. For comparison, pure gold and silver nanoparticles were synthesized with the same reaction parameters.

**Figure 1:**
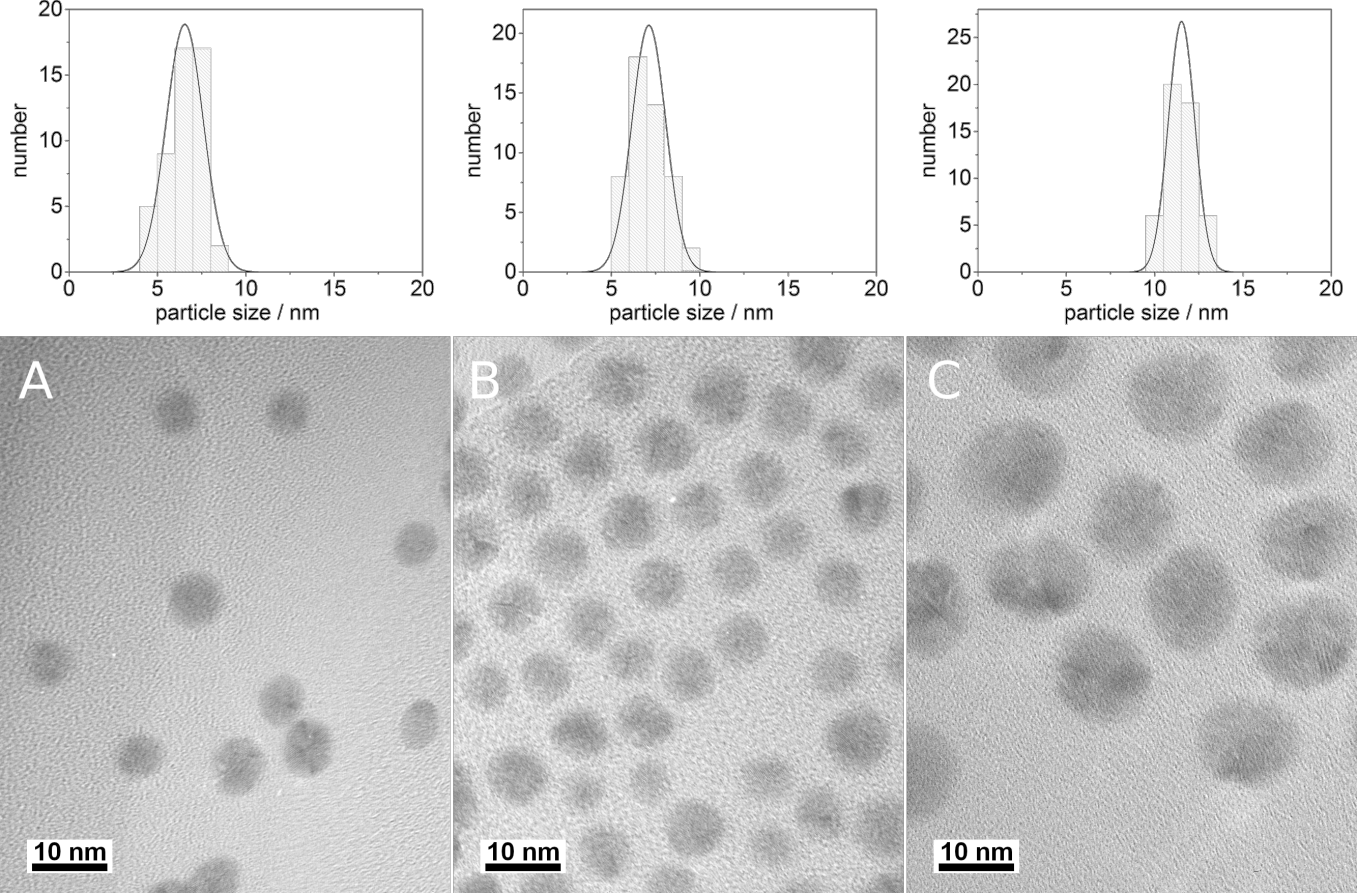
Transmission electron micrographs of PVP-functionalized Ag/Au nanoparticles: (A) Ag/Au 10:90, *d* = 6.5 nm; (B) Ag/Au 30:70, *d* = 7.1 nm; (C) Ag/Au 90:10, *d* = 11.5 nm, with the particle size distribution shown in the histograms.

The size and morphology of the nanoparticles were determined by transmission electron microscopy (TEM), differential centrifugal sedimentation (DCS) and dynamic light scattering (DLS). TEM showed that the nanoparticles were nearly monodisperse, quasi-spherical, polycrystalline, and had a uniform diameter of 6 to 7 nm. Only nanoparticles with the highest silver content (Ag/Au 90:10) had a slightly larger diameter (≈11 nm). This trend was maintained by pure silver particles which showed a considerably larger diameter when synthesized with the same reaction parameters (≈30 nm). The TEM images in Figure 1 show a representative series of Ag/Au 10:90, 30:70, and 90:10 nanoparticles with PVP stabilization.

**Figure 2:**
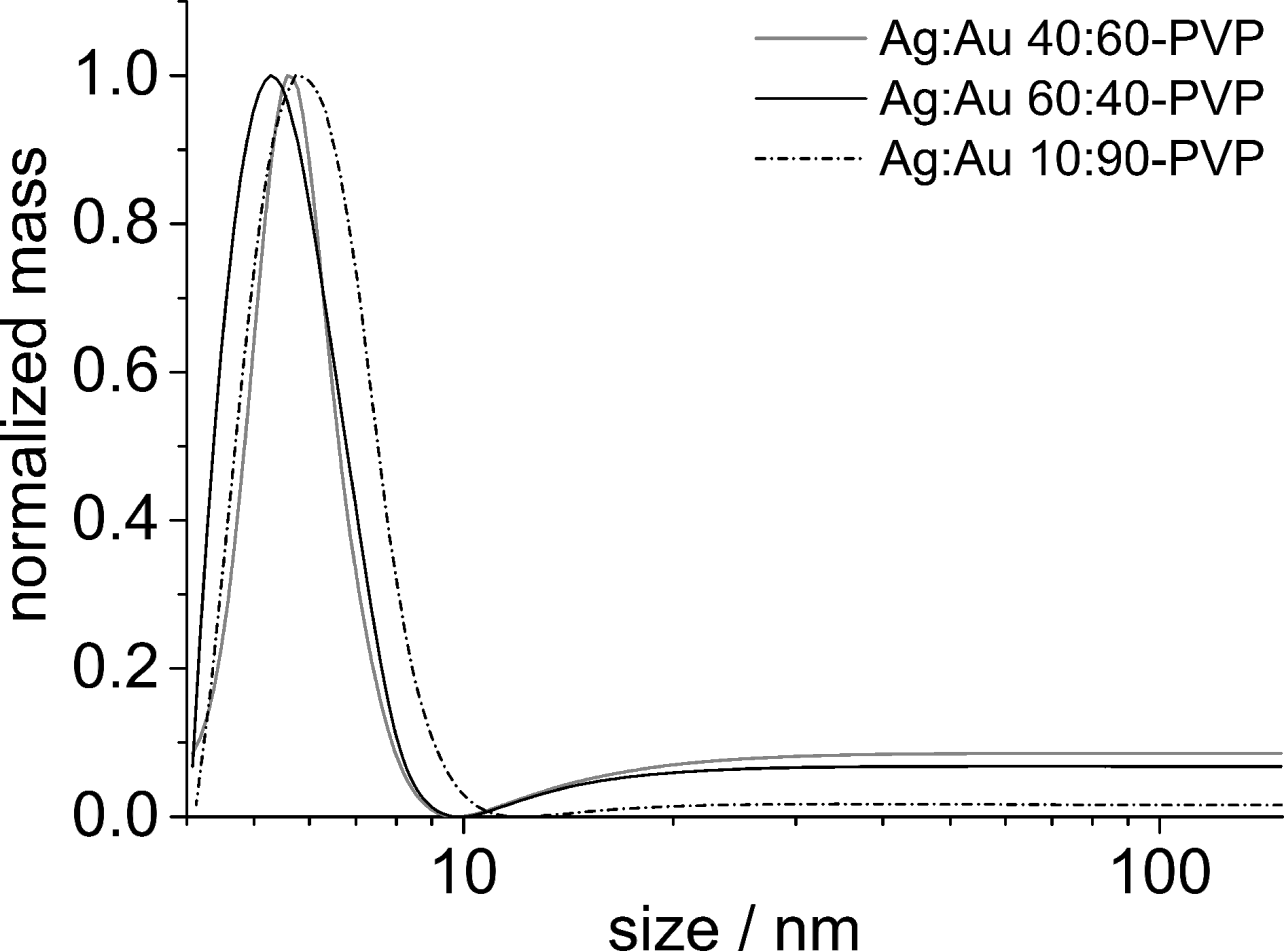
DCS results of Ag/Au–PVP nanoparticles of three different compositions: Ag/Au 40:60, *d* = 5.3 nm; Ag/Au 60:40, *d* = 5.5 nm; Ag/Au 10:90, *d* = 5.8 nm.

TEM alone cannot be used to determine the dispersion of nanoparticles in solution [33–35]. DCS analysis showed that the purified samples did not contain any agglomerates and maintained a high degree of monodispersity. The nanoparticles showed a narrow size distribution with an average particle size of ≈6 nm, except for the Ag/Au 90:10 nanoparticles that exhibited an average diameter of 8 nm. Figure 2 shows representative DCS graphs of samples of three different compositions after ligand exchange with PVP.

Dynamic light scattering also showed a monomodal particle size distribution without agglomerates. The polydispersity index between 0.1 and 0.3 confirmed a good degree of monodispersity. Note that the hydrodynamic radius, *d*_H_, as probed by DLS, is slightly larger (10–12 nm) than the radius determined by DCS or TEM (see [30,34] about this systematic difference), but independent of the particle composition. Only the pure Ag nanoparticles had a much larger hydrodynamic radius of 22 nm. Analysis of the electrophoretic mobility yielded negative zeta potentials, indicating a reasonable electrostatic stability of the particles. Note that the variation of the zeta potential is probably within the range of the experimental noise. Table 1shows all size-related data of the alloyed nanoparticles. The experimental molar compositions of the nanoparticles were examined by AAS. The silver and gold values were close to the theoretical compositions (Table 2).

**Table 1:** Results of nanoparticle diameters determined from TEM, DCS and DLS (by number) and zeta potential measurements of PVP-functionalized Ag/Au nanoparticles as well as for pure Ag and Au nanoparticles. The given errors represent standard deviations.

Ag/Au / mol %:mol %	*d* (TEM) / nm	*d* (DCS) / nm	*d*_H_ (DLS) / nm	Zeta potential / mV

0:100	6.0 ± 0.7	4.8	9 ± 2	−31 ±13
10:90	6.5 ± 1.1	5.5	10 ± 3	−33 ±11
20:80	6.9 ± 1.0	5.4	10 ± 3	−47 ±7
30:70	7.1 ± 1.0	5.5	12 ± 4	−31 ± 12
40:60	7.0 ± 0.5	5.6	10 ± 2	−23 ± 8
50:50	6.9 ± 0.7	5.7	10 ± 3	−25 ± 9
60:40	7.1 ± 0.9	5.5	11 ± 3	−39 ± 8
70:30	8.5 ± 1.0	5.3	11 ± 2	−39 ± 10
80:20	8.5 ± 1.0	6.8	12 ± 2	−42 ± 7
90:10	11.5 ± 0.7	8.1	12 ± 4	−33 ± 9
100:0	34 ± 6	29	22 ± 9	−22 ± 9

**Table 2:** Experimental molar composition of the Ag/Au nanoparticles as measured by AAS.

Theoretical molar Ag/Au composition	Molar Ag/Au composition from AAS

10:90	8:92
20:80	17:83
30:70	29:71
40:60	37:63
50:50	49:51
60:40	58:42
70:30	68:32
80:20	78:22
90:10	87:13

**Figure 3:**
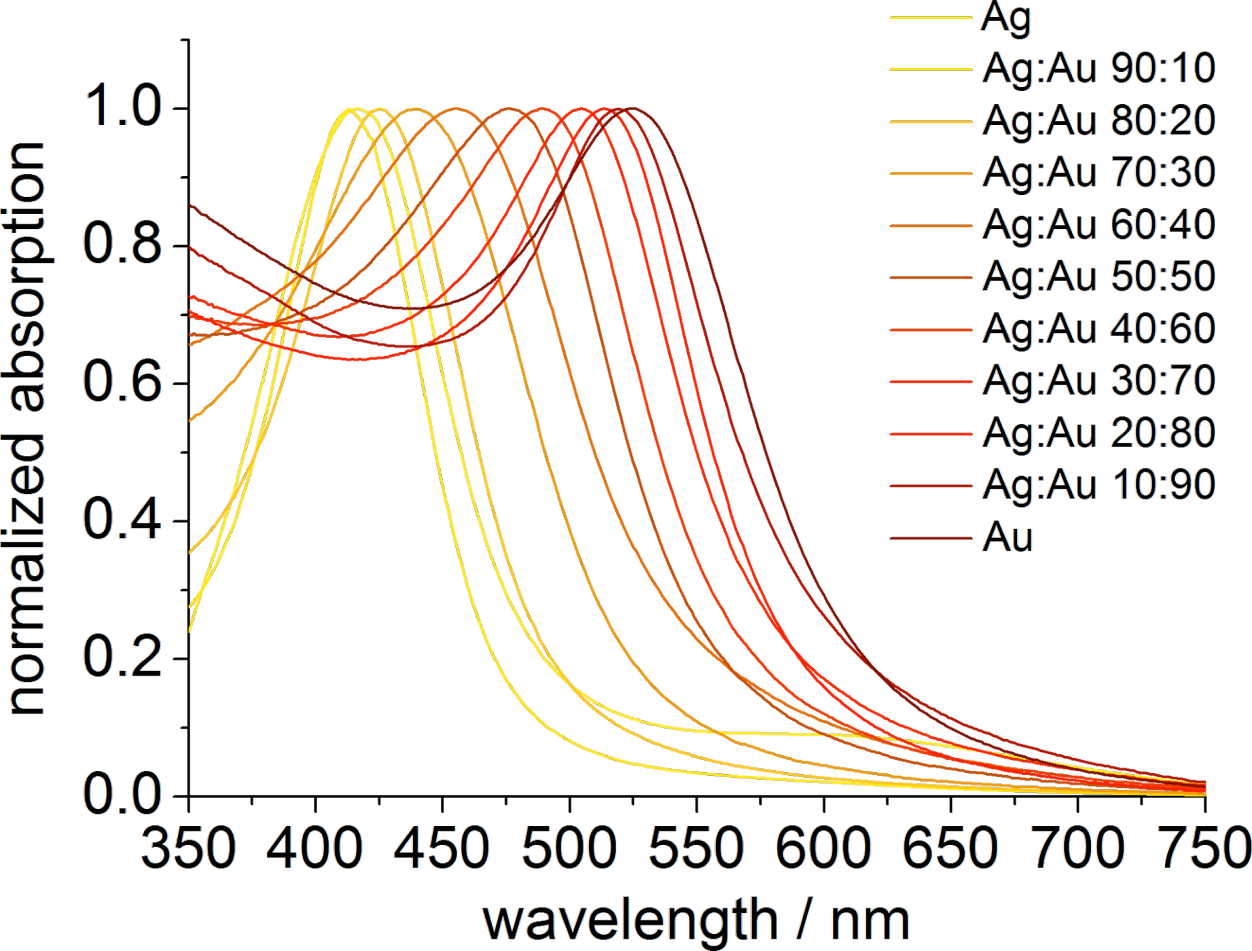
UV–vis spectra of PVP-functionalized Ag/Au nanoparticles and pure Ag and Au nanoparticles.

To confirm the alloying of the two metals, UV–vis spectra were recorded for all samples. From the spectra it is possible to gain information about the inner structure of the nanoparticles. In case of alloyed Ag/Au nanoparticles, the plasmon resonance peak shows one maximum due to the distribution of the metals throughout the whole particle. Core–shell nanoparticles or individual silver or gold nanoparticles show two distinct plasmon resonance peaks [21,36–37]. As it is depicted in Figure 3, the absorption spectra show only one narrow peak with a maximum absorption wavelength dependent on the silver/gold ratio, indicating the formation of nanoalloys.

**Figure 4:**
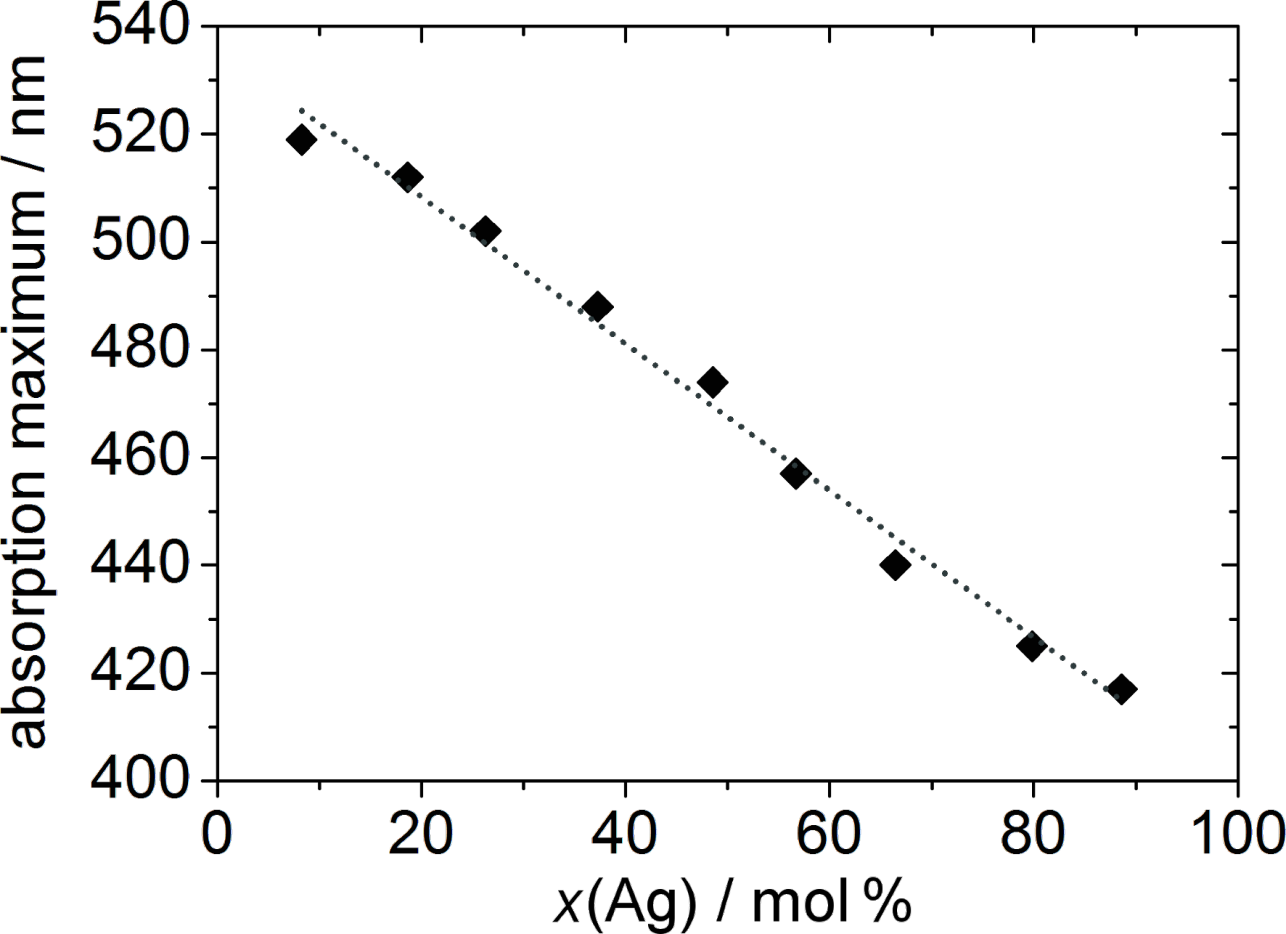
Overview of the absorption maxima in UV–vis spectroscopy of PVP-functionalized Ag/Au nanoparticles as function of the experimentally determined silver molar content (Table 2).

A simple method to examine the overall distribution of the metals (not in individual particles, but rather in the sample as a whole) is to plot the maximum absorption wavelength of the plasmon resonance spectra against the molar fraction of Au or Ag. For a given particle size and surface functionalization, a linear relationship would indicate a macroscopically homogeneous distribution of the metals in the nanoparticles [21]. In Figure 4, the absorption maxima of the ≈10 nm nanoparticles obtained from the standard synthesis protocol are shown. The trend is almost linear, suggesting a good homogeneity of the alloyed metals, although some gradient in the composition within the nanoparticle cannot be ruled out. However, a core–shell structure with distinct, separate silver and gold regions can be verified.

### Cell culture experiments

To examine the cytotoxicity with regards to the molar fraction of silver in the nanoparticles, HeLa cells and human mesenchymal stem cells were incubated with nanoalloys of nine different compositions and also with pure gold and pure silver nanoparticles. In order to compare the samples, the total amount of metal was chosen as the fixed parameter. The actual nanoparticle concentration, shown in Table 3, was calculated on the basis of the measured nanoparticle diameter from DCS and the assumption that the particles are perfectly spherical and monodisperse.

**Table 3:** Calculated nanoparticle (NP) concentration for cell viability experiments.

Theoretical Ag/Au composition / mol %	NP conc. at 5 µg mL^−1^ metal / pmol mL^−1^	NP conc. at 50 µg mL^−1^ metal / pmol mL^−1^	NP conc. at 100 µg mL^−1^ metal / pmol mL^−1^

0:100	0.5	5.5	11
10:90	5.0	50	101
20:80	4.8	48	96
30:70	4.0	40	80
40:60	5.9	59	118
50:50	4.1	41	82
60:40	6.3	63	126
70:30	4.2	42	83
80:20	3.6	36	73
90:10	4.3	43	86
100:0	6.6	66	132

**Figure 5:**
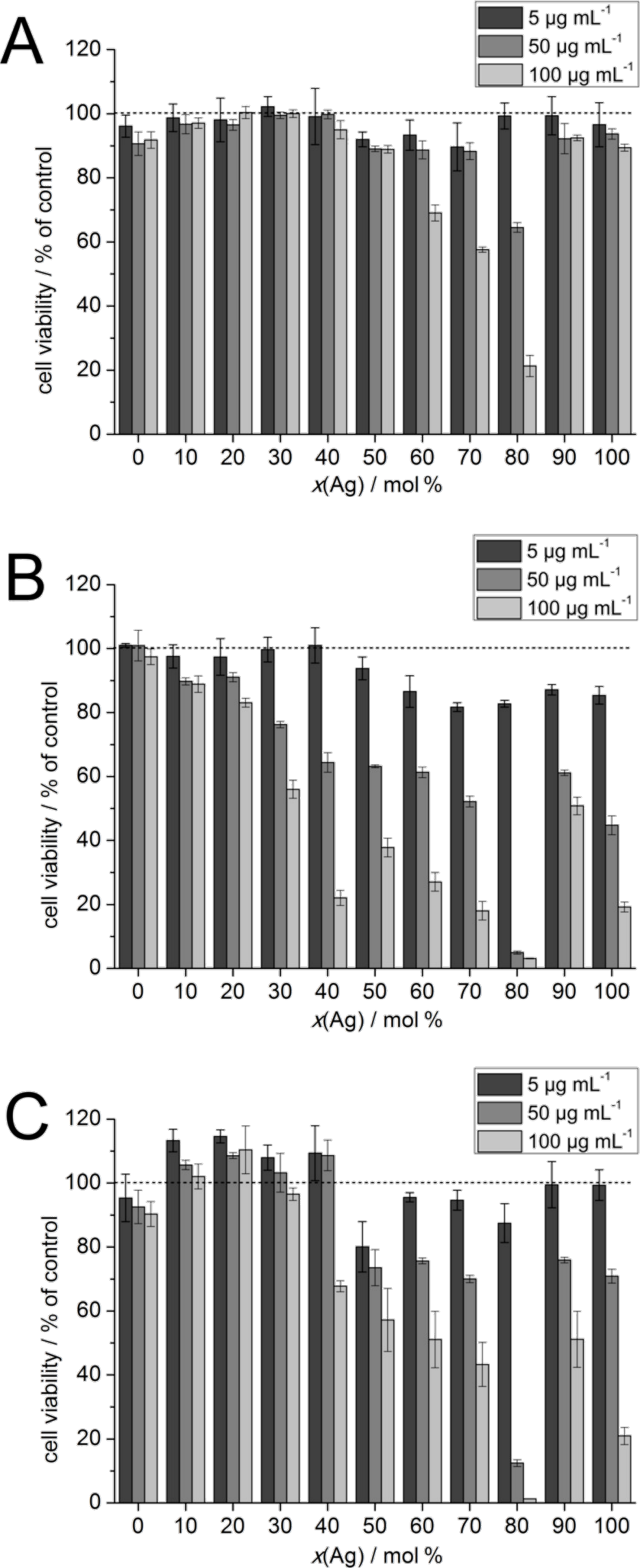
The viability of HeLa cells after incubation with alloyed nanoparticles and pure silver and gold nanoparticles according to the nominal silver content. The experiments were carried out at total metal concentrations of 5, 50, and 100 µg mL^−1^. The cytotoxicity tests were performed at (A) 5 h, (B) 24 h, (C) and 72 h after the nanoparticle addition. The dotted lines indicate the viability of the control (untreated cells).

**Figure 6:**
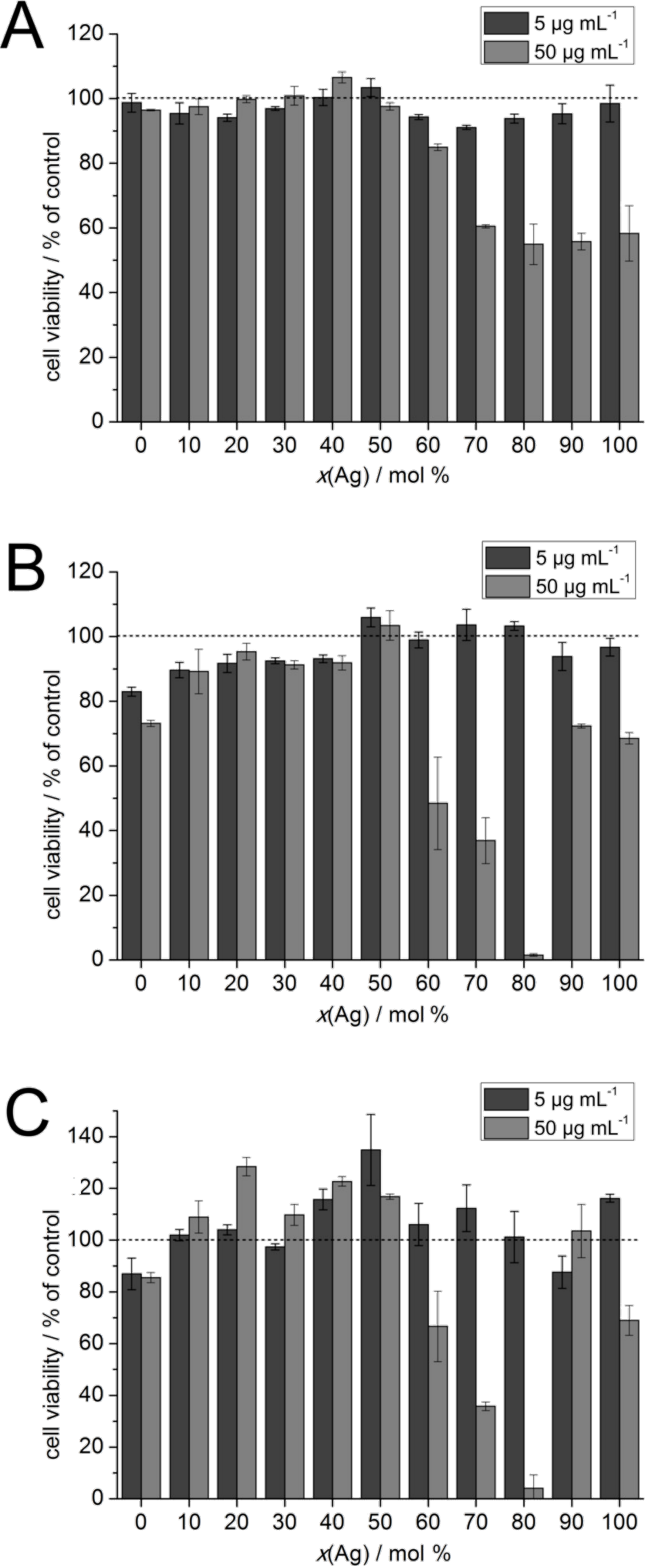
The viability of hMSCs after incubation with alloyed nanoparticles and pure silver and gold nanoparticles according to the nominal silver content. The experiments were carried out at total metal concentrations of 5 and 50 µg mL^−1^. The cytotoxicity tests were performed at (A) 5 h, (B) 24 h, (C) and 72 h after the nanoparticle addition. The dotted lines indicate the viability of the control (untreated cells).

Figure 5 and Figure 6 show the viability of HeLa cells and hMSCs after their treatment with the nanoparticles according to the MTT test. As was expected, the cytotoxicity of the nanoparticles increased with increasing silver content. Moreover, the toxicity of the nanoparticles was concentration-dependent and increased with longer incubation time.

The relationship between silver content and toxicity was not linear. After 5 h, only the highest metal concentrations, namely 100 µg mL^−1^ for HeLa cells and 50 µg mL^−1^ for hMSCs, showed significant effects on the cells. Interestingly, only the nanoparticles with a molar silver composition of 60% or higher affected the cell viability. After 24 h, this trend was observed more clearly. For HeLa cells, toxic effects began to emerge for nanoparticles with a silver content >30 mol % and also at a metal concentration of 50 µg mL^−1^.

## Discussion

The synthesis presented herein is well suited for the generation of monodisperse, bimetallic, Ag/Au nanoparticles of different molar compositions. The particles can be coated with a polymer shell (e.g., PVP) for enhanced colloidal stability in aqueous medium. The characterization of the nanoparticles using complementary analytical methods showed uniform particle sizes independent of the molar metal composition. Only nanoparticles with the highest silver content (Ag/Au 90:10) showed a slightly larger particle size. This can likely be attributed to the comparatively slow reduction process and the different standard potentials of the two noble metals. Silver, as the less noble metal, is reduced more slowly than gold, leading to a slower nucleation and larger particle diameter for high silver content. Compared to the TEM images, the DCS measurements resulted in smaller nanoparticle diameters. This is due to a slightly smaller effective density of the particle due to the polymer shell and is consistent with previous results [30].

Prior to the in vitro experiments, multiple ultracentrifugation steps were employed to ensure a comprehensive purification of the samples. Regarding the toxicity towards HeLa cells and hMSCs, it was generally found that the toxicity of the alloyed silver gold nanoparticles with a silver content up to 50 mol % was not as high as anticipated with respect to the relative silver concentration. This is probably caused by an alloying effect in which the gold somehow passivates the silver and reduces the amount of released silver ions. In a comparable toxicity study with laser-generated alloyed Ag/Au nanoparticles on cumulus-oocyte complexes and spermatozoa [38] and human gingival fibroblasts [39], a passivating effect of gold on silver was reported. In contrast to these studies, the toxicity our nanoalloys reached a maximum toxicity for Ag/Au 80:20 nanoparticles. Both HeLa cells and hMSC were almost quantitatively killed at the highest metal concentrations of Ag:Au 80:20 nanoparticles, while the Ag/Au 90:10 nanoparticles and also the pure silver nanoparticles were much less toxic.

This can be ascribed to the slightly different particle size and a likely inhomogeneous distribution of silver and gold throughout the particle. The comparably lower toxicity of the silver nanoparticles can be partially attributed to the slightly larger size of the nanoparticles. Due to the larger diameter and lower specific surface area, the pure silver nanoparticles should release silver ions at a lower rate. Furthermore, cells treated with nanoparticles that contain more gold than silver remained viable after 72 h. This increase in viability by addition of gold containing nanoparticles to the cells was also reported by Mahl et al. [30].

When investigations about cellular and bacterial toxicity are carried out, the purification of the nanoparticles is a crucial factor. As silver containing nanoparticles are often prone to release silver ions during storage that are more toxic than the nanoparticles themselves [9,40–41], it is important to separate the toxic effects of the nanoparticles and unreacted material from the synthesis [11,42]. As some reported cell culture experiments with alloyed silver–gold nanoparticles were conducted without purification of the dispersions [19,43–44], it cannot be ruled out for these cases that the toxic effects were not only caused by the nanoparticles but also by synthesis byproducts (e.g., unreacted silver ions).

Alloyed silver–gold nanoparticles have cytotoxic effects that are not proportional to the silver content. Li et al. found an increased toxicity towards *Daphnia magnia* of citrate-stabilized alloyed nanoparticles with high gold content (Ag/Au 20:80) and a decreased toxicity of nanoparticles with high silver content (Ag/Au 80:20) with respect to the expected toxicity based on the silver content [43]. Tiedemann et al. and Grade et al. reported a decrease in the silver-induced toxicity with increasing gold fractions in alloyed silver–gold nanoparticles [25,27,38]. The nanoparticles used in these experiments were laser-generated alloys that had a uniform distribution of silver and gold over the whole particle. On cumulus-oocyte complexes and spermatozoa, the nanoparticles showed toxic effects when the molar silver content was higher than 50%. Still, the effect was lower than the expected toxicity based on the silver content [38]. Similar results were found for human gingival fibroblasts and *S. aureus* [25]. For our investigations on HeLa cells and hMSCs, we used 6 to 7 nm silver–gold nanoalloys prepared by wet chemistry, having a slightly higher gold content in the core [30,45]. Nevertheless, the observed toxic effects resembled the findings of Tiedemann et al. and Grade et al. with an unexpectedly low toxicity for nanoparticles with a molar silver content of less than 50% [25,38]. Notably, the samples with a molar silver/gold fraction of 80:20 showed a very high toxicity that was greater than that of pure silver nanoparticles.

## Conclusion

Alloyed silver–gold nanoparticles of nine different compositions were prepared with a versatile and facile wet chemical synthesis that allows size control and ligand exchange without affecting the resulting nanoparticle purity or stability. The in vitro studies were performed using HeLa cells and hMSCs. The cytotoxicity increased with increasing silver content in the nanoalloys, but the observed effect is not proportional to the relative silver amount. While nanoparticles with a silver content less than 40 mol % do not show any cytotoxicity, nanoalloys with silver contents of 40 mol % and 50 mol % show intermediate toxic effects from which the cells can partially recover. It is possible that a passivating effect from the alloyed gold is responsible for these observations. Future studies on the time-dependent dissolution of such alloyed nanoparticles in biological media may help to better understand this effect.

## Experimental

### Chemicals

We used silver nitrate (Roth, p.a.), trisodium citrate dihydrate (AppliChem, p.a.), tannic acid/tannin (Acros, 95%, *M*_w_ = 1,701.23 g mol^−1^, C_76_H_52_O_46_), poly(vinylpyrrolidone) (PVP K 30, Povidone 30; Fluka, *M*_w_ = 40,000 g mol^−1^), and tris(3-sulfonatophenyl)phosphine hydrate, sodium salt (10–15% oxide) (Strem Chemicals). HAuCl_4_ was prepared by dissolution of gold in aqua regia according to standard procedures. Ultrapure water (Purelab ultra instrument from ELGA) was used for all preparations.

### Synthesis

Prior to use, all glassware was cleaned with boiling aqua regia. The nanoparticles were synthesized by reduction with citrate and tannic acid in aqueous media similar to previously described procedures [30] with slight modifications. In particular, we used higher amounts of the reducing agent (15 mg citrate and 4.2 mg tannic acid) to generate smaller nanoparticles with a mean diameter of 6 to 7 nm.

50 mL of degassed ultrapure water was heated to 100 °C. A cumulative volume of 500 µL of 10 mM solutions of HAuCl_4_ and AgNO_3_ (total metal content of 5 µmol), depending on the intended composition of the nanoparticles, was added. A mixture of 0.75 mL of 2 wt % trisodium citrate and 1.5 mL of a 2.8 mg mL^−1^ tannic acid solution was quickly added with a pipette under vigorous stirring. The reaction mixture was stirred for 10 min.

Further functionalization was carried out by adding 1 mL of a 10 mg mL^−1^ PVP solution to the unpurified dispersion. After stirring for at least two more hours, the nanoparticles were separated from the unreacted material by ultracentrifugation (30,000 rpm, 60,000*g*, 30 min) and redispersed in ultrapure water with a vortex. The purification step was repeated twice. The synthesis was scaled up by a factor of ten without any noticeable effect on the properties of the nanoparticles.

### Characterization

Transmission electron microscopy (TEM) images were recorded with a Philips CM 200 FE instrument. The dispersions were diluted with deionized water, drop cast onto a carbon-coated copper grid and dried under ambient conditions. The particle diameter was estimated by manually measuring 50 particles and compiling a histogram. Differential centrifugal sedimentation (DCS) was performed with a CPS Instruments DC 24000 disc centrifuge (24,000 rpm). Two sucrose solutions (8 wt % and 24 wt %) formed a density gradient which was capped with 0.5 mL dodecane as a stabilizing agent. The calibration standard was a poly(vinylchloride) (PVC) latex in water with a particle size of 476 nm provided by CPS Instruments. The calibration was carried out prior to each run. A sample volume of 100 µL was used. Dynamic light scattering (DLS) was carried out on a Malvern Zetasizer Nano ZS ZEN 3600 instrument (25 °C, laser wavelength 633 nm). The scattering was monitored at a fixed angle of 173° in backward scattering mode. The primary data were derived from the correlation function of the scattered intensity as a number-weighed size distribution. UV–vis spectroscopy was performed with a Varian Cary 300 instrument. Suprasil^®^ cuvettes with a sample volume of 3.5 mL were used. Atomic absorption spectroscopy (AAS) was carried out with a Thermo Electron M-Series spectrometer with a graphite tube furnace according to DIN EN ISO/IEC 17025:2005 after dissolving the particles in aqua regia.

### Cell culture

HeLa cells (human transformed cervix epithelial cells) and human mesenchymal stem cells (hMSCs) were used for cell experiments. The HeLa cells were cultured in DMEM (Dulbecco's Modified Eagle's Medium), supplemented with 10% of fetal bovine serum (FBS), 100 U mL^−1^ penicillin, and 100 U mL^−1^ streptomycin. The hMSCs were cultivated in RPMI 1640, containing 10% FBS, 100 U mL^−1^ penicillin, 100 U mL^−1^ streptomycin, 2 mM L-glutamine and 10 mM HEPES. The cells were incubated at 37 °C in a humidified atmosphere with 5% CO_2_. Approximately 12 h before the addition of the nanoparticles, the cells were trypsinized and seeded in 24-well plates with a density of 2.5·10^4^ and 2.0·10^4^ cells per well for HeLa cells and hMSCs, respectively.

The cytotoxicity tests were performed after 5 h, 24 h and 72 h of incubation with nanoparticles (nine samples of different Ag/Au molar ratios, pure silver nanoparticles, and pure gold nanoparticles). 500 µL of the nanoparticles suspension were added per well. The HeLa cells were incubated with 5, 50 or 100 µg of metal (silver and/or gold) per mL of dispersion. For the hMSCs, we used 5 µg mL^−1^ and 50 µg mL^−1^ metal concentrations. The total amount of metal in the alloyed nanoparticles was given as a sum of silver and gold, determined by atomic absorption spectroscopy.

The cell viability was analyzed by the MTT assay. 3-(4,5-dimethylthiazol-2-yl)-2,5-diphenyl-2*H*-tetrazolium bromide (MTT; Sigma, Taufkirchen, Germany) was dissolved in PBS (5 mg mL^−1^) and then diluted to 1 mg mL^−1^ in the cell culture medium. After incubation, the cell culture medium with nanoparticles was replaced by 300 µL of the MTT solution for 1 h. Then, the MTT solution was replaced by 300 µL of DMSO and incubated at 37 °C under 5% CO_2_ in a humidified atmosphere. After 30 min, a 100 µL aliquot was taken for spectrophotometric analysis with a Multiscan FC instrument (Thermo Fisher Scientific, Vantaa, Finland) at λ = 570 nm. The absorption spectra of the cells was normalized to that of control cells (incubated without nanoparticles), thereby giving the relative level of cell death. A live–dead viability/cytotoxicity assay (Calcein AM, ethidium homodimer III) was also carried out, but the data were not included because it was impossible to obtain reproducible results. The presence of nanoparticles did not permit a reliable quantitative analysis.
